# Erythromelalgia in an Adolescent Female

**DOI:** 10.31138/mjr.33.2.256

**Published:** 2022-06-30

**Authors:** Eleftheria Mamatsi, Melpomeni Giorgi, Argirios Dinopoulos, Vasiliki Papaevangelou, Lampros Fotis

**Affiliations:** Department of Pediatrics, Attikon General Hospital, National and Kapodistrian University of Athens, Greece

**Keywords:** amitriptyline, aspirin, carbamazepine, child, erythromelalgia

## Abstract

Erythromelalgia is a disabling syndrome of paroxysmal vasodilation affecting the feet, hands and face characterised by patient’s cooling behaviour to achieve symptom relief. It can be primary or secondary and although a rare disorder it has been described in children and adolescents. We describe the case of a 14-year-old female diagnosed with primary erythromelalgia successfully treated with aspirin, amitriptyline, and carbamazepine.

## CASE PRESENTATION

**Figure 1.**
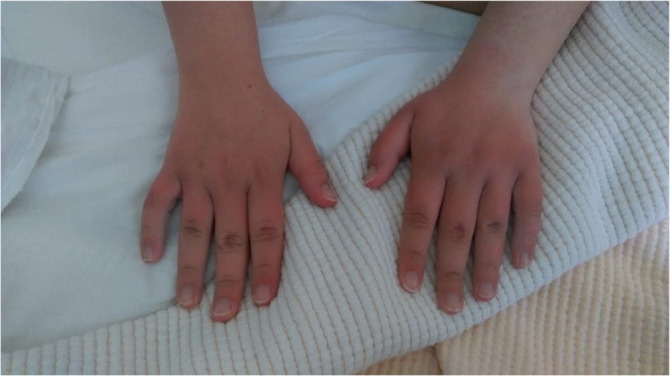
Redness and oedema of fingers and dorsal hands.

**Figure 2.**
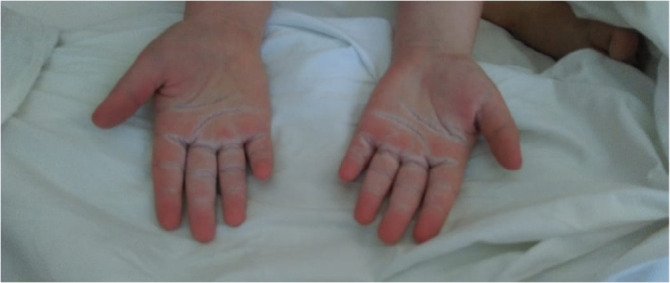
Maceration of the skin of the palm.

**Figure 3.**
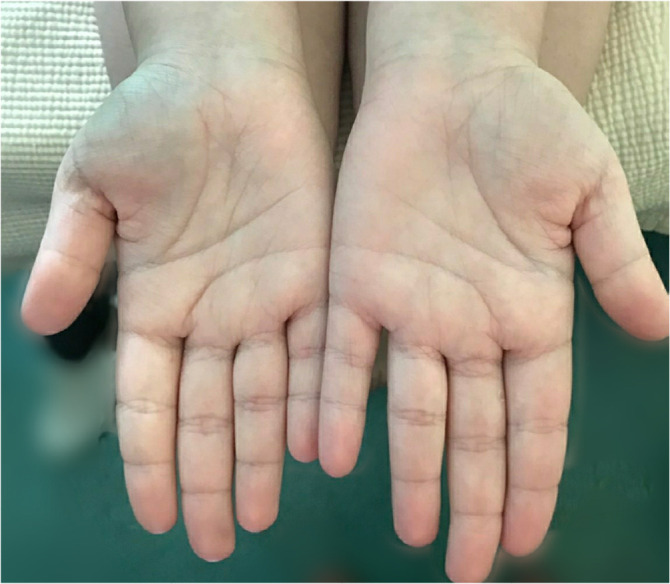
Dorsal hands 15 days after treatment initiation.

**Figure 4.**
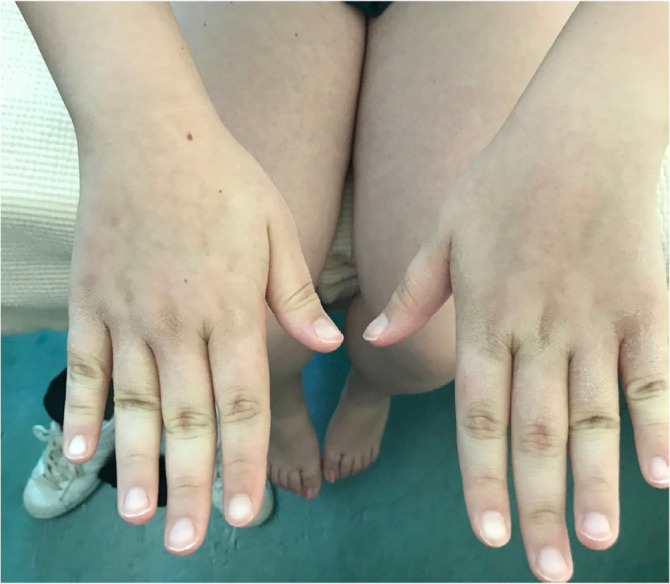
Normal palm skin 15 days after treatment initiation.

A 14-year-old female was referred due to burning pain of her feet followed 2 weeks later by similar symptoms in her hands, for the last 6 weeks. She was previously treated with non-steroidal anti-inflammatory drugs and oral steroids with no improvement. Her physical examination revealed redness and oedema of the dorsal surface of the fingers, hands and palms, in both hands (**Figure 1**), with erosion of the skin of the palms due to continuous immersion to water (**Figure 2**). She had no evidence of arthritis, nor any other signs suggestive of a systemic inflammatory disorder. Laboratory testing revealed white blood count 6550 K/μl, haemoglobin 13,4 g/dl, platelets 308 K/μl, erythrocyte sedimentation rate 18 mm/1h, C-reactive protein 0.1 mg/L, AST 11 U/L, ALT 6 U/L, CPK 27 U/L, C3 91 mg/dl and C4 21 mg/dl. Antinuclear antibodies, rheumatoid factor, cyclic citrullinated peptide antibody, antineutrophil cytoplasmic antibodies, lupus anticoagulant, β2 – glycoprotein IgG and IgM, anti-cardiolipin antibodies IgG and IgM were negative. Genetic testing for the *SCN9A* gene mutation, which encodes the voltage-gated sodium channel Na(v)1.7 was negative. Often, a gain of function mutation of this gene is associated both with familial and sporadic cases of erythromelalgia.^1^ The diagnosis of primary erythromelalgia was established and managed with behavioural interventions (limb elevation, cooling techniques with fan and water for 10 min per hour) and per os Aspirin (200 mg/day), Carbamazepine (200 mg/day), Amitriptyline (25 mg/day), and Clobazam (30 mg/day), all initiated simultaneously. Two weeks after starting treatment her symptoms were controlled and her sleep was fully restored. The hand skin healed completely, except for mild dry skin on the dorsal surface (**Figure 3** and **Figure 4**).

## DISCUSSION

Erythromelalgia has an incidence of less the 2 per 100000 people per year. It is more common in women than in men and exceedingly rare in children.^2–4^ It usually affects the feet (88%) and less commonly the hands (25%), legs (23%), face, ear, and nose.^4^ Symptoms are often symmetrical and during episodes the affected areas become red, hot, and painful, while swelling occasionally occurs.^3^ A characteristic feature of erythromelalgia is patient’s cooling behaviour to achieve symptom relief. Potential complications of cooling techniques include “windburn” from fans, and signs and symptoms of frostbite secondary to overuse of ice. Chronic immersion of the extremity in water may lead to skin maceration, oedema, and ulcerations as in the case presented.^5^ The majority of both familial and sporadic cases of primary erythromelalgia are attributed to gain-of-function mutations of SCN9A*,* which encodes the voltage-gated sodium channel Na(v)1.7.^1^ Secondary erythromelalgia has been associated with thrombocytosis in children, but not with other myeloproliferative disorders as described in adults, while it can also develop in patients with small fibre neuropathies, autoimmune disorders, malignancies, multiple sclerosis, hypercholesterolemia, hypertension obesity, type 1 diabetes mellitus, and heavy-metal poisoning.^3,6,7^

There is no cure for erythromelalgia and combination of non-pharmacological and pharmacological interventions such as local treatment, aspirin and anti-epileptics are directed towards improving quality of life and reducing symptoms.^4,8^

## References

[1] Cregg R Laguda B Werdehausen R Cox JJ Linley JE Ramirez JD Novel mutations mapping to the fourth sodium channel domain of nav1.7 result in variable clinical manifestations of primary erythromelalgia. NeuroMolecular Medicine 2013;15:265–78. 23292638 10.1007/s12017-012-8216-8 PMC3650253

[2] Reed KB Davis MDP. Incidence of erythromelalgia: A population-based study in Olmsted County, Minnesota. Journal of the European Academy of Dermatology and Venereology 2009;23:13–5. 18713229 10.1111/j.1468-3083.2008.02938.x PMC2771547

[3] Cook-Norris RH Tollefson MM Cruz-Inigo AE Sandroni P Davis MDP Davis DMR. Pediatric erythromelalgia: A retrospective review of 32 cases evaluated at Mayo Clinic over a 37-year period. J Am Acad Dermatol 2012;66:416–23. 21798623 10.1016/j.jaad.2011.01.010

[4] Davis MDP O’Fallon WM Rogers RS Rooke TW. Natural history of erythromelalgia: Presentation and outcome in 168 patients. Arch Dermatol 2000;136:330–6. 10724194 10.1001/archderm.136.3.330

[5] Davis MDP. Immersion foot associated with the overuse of ice, cold water, and fans: A distinctive clinical presentation complicating the syndrome of erythromelalgia. J Am Acad Dermatol 2013;69:169–71. 23768296 10.1016/j.jaad.2013.02.021

[6] Layzer RB. Hot feet: Erythromelalgia and related disorders. J Child Neurol 2001;16:199–202. 10.1177/088307380101600307 11305688

[7] Drenth JPH Michiels JJ Özsoylu S. Acute secondary erythermalgia and hypertension in children. Eur J Pediatr 1995;154:882–5. 8582398 10.1007/BF01957497

[8] Natkunarajah J Atherton D Elmslie F Mansour S Mortimer P. Treatment with carbamazepine and gabapentin of a patient with primary erythermalgia (erythromelalgia) identified to have a mutation in the SCN9A gene, encoding a voltage-gated sodium channel. Clin Exp Dermatol 2009;34:e640–2. 19549232 10.1111/j.1365-2230.2009.03355.x

